# Cytotoxicity and reactive oxygen species production induced by different co-monomer eluted from nanohybrid dental composites

**DOI:** 10.1186/s12903-023-02710-y

**Published:** 2023-01-30

**Authors:** En-Shi Jiang, Wonjoon Moon, Bum-Soon Lim, Juhea Chang, Shin Hye Chung

**Affiliations:** 1 Department of Dental Biomaterials Science, School of Dentistry and Dental Research Institute, Seoul National University, 101 Daehak-Ro, Jongno-Gu, Seoul, 03080 Republic of Korea; 2 Department of Stomatology, Yanbian University and Affiliated Hospital of Yanbian University, Yanji, 133000 China; 3 Harvard Medical School, Boston, MA 02115 USA; 4 Wellman Center for Photomedicine, Massachusetts General Hospital, Boston, MA 02114 USA; 5 National Dental Care Center for Persons With Special Needs, Seoul National University Dental Hospital, 101 Daehak-Ro, Jongno-Gu, Seoul, 03080 Republic of Korea

**Keywords:** Nanohybrid, Dental composites, Eluates, Cytotoxicity, Reactive oxygen species, Gas chromatography/mass spectrometry

## Abstract

**Background:**

Safety issues for dental restorative composites are critical to material selection, but, limited information is available to dental practitioners. This study aimed to compare the chemical and biological characteristics of three nanohybrid dental composites by assessing filler particle analysis, monomer degree of conversion (DC), the composition of eluates, and cytotoxicity and reactive oxygen species (ROS) production in fibroblasts.

**Methods:**

Three nanohybrid composites (TN, Tetric N-Ceram; CX, Ceram X Sphere Tec One; and DN, DenFil NX) were used. The size distribution and morphology of the filler particles were analysed using scanning electron microscopy (*n* = 5). The DC was measured via micro-Raman spectroscopy (*n* = 5). For the component analysis, methanol eluates from the light-polymerised composites were evaluated by gas chromatography/mass spectrometry (*n* = 3). The eluates were prepared from the polymerised composites after 24 h in a cell culture medium. A live/dead assay (*n* = 9) and Water-Soluble Tetrazolium-1 assay (*n* = 9) were performed and compared with negative and positive controls. The ROS in composites were compared with NC. Statistical significance in differences was assessed using a t-test and ANOVA (*α* = 0.05).

**Results:**

Morphological variations in different-sized fillers were observed in the composites**.** The DC values were not significantly different among the composites. The amounts of 2-hydroxyethyl methacrylate (HEMA) were higher in TN than DN (*p* = 0.0022) and triethylene glycol dimethacrylate (TEGDMA) in CX was higher than in others (*p* < 0.0001). The lowest cell viability was shown in CX (*p* < 0.0001) and the highest ROS formation was detected in TN (*p* < 0.0001).

**Conclusions:**

Three nanohybrid dental composites exhibited various compositions of filler sizes and resin components, resulting in different levels of cytotoxicity and ROS production. Chemical compositions of dental composites can be considered with their biological impact on safety issues in the intraoral use of dental restorative composites. CX with the highest TEGDMA showed the highest cytotoxicity induced by ROS accumulation. DN with lower TEGDMA and HEMA presented the highest cell viability.

**Supplementary Information:**

The online version contains supplementary material available at 10.1186/s12903-023-02710-y.

## Background

The wide-ranged applications of resin-based composites in restorative dentistry facilitated the improvement of the mechanical and aesthetic features as well as clinical performance. To enhance the material properties, various technologies have been introduced, mainly those dealing with changes in filler components and monomer-matrix formulation [1–4]. Among restorative composite resins with various filler distributions, nanohybrid composites incorporate both nano-ranged sizes of inorganic fillers (0.005–0.01 μm) and microsized fillers (0.01–0.04 μm) [5]. The nano-sized fillers, are smaller than the visible light wavelengths and occupy the spaces between larger particles. Additional filler loading by submicron-sized particles led to improved surface qualities such as superior polish and gloss retention compared to conventional micro-hybrid composites [6, 7]. However, small sizes of filler particles increase the surface area-to-volume ratios of the fillers, which may make the polymerised structures prone to water uptake and induce interfacial degradation of the resin matrix and filler particles [8]. In addition, water absorption and moisture permeation into the pores within the incompletely regarding the monomers and additives released from resin-based composites leaching into the oral environment in diverse polymerizing conditions [11–13]. Cell death caused by DNA double-strand breakage, alveolar bone resorption by increased inflammatory cytokine activity, the inflammatory reaction by increased COX-2 enzyme, and acute systemic toxicity are significant concerns of leached components [14–17]. Bisphenol-A (BPA) is a well-known endocrine disruptor that can be present as an impurity or degradation product of BPA-based monomers [18]. Co-monomers of low molecular weight, such as triethylene glycol dimethacrylate (TEGDMA) and 2-hydroxyethyl methacrylate (HEMA), are more mobile and absorbent and readily leach into the immersion medium relative to basic monomers with high molecular weights, such as bisphenol A glycidyl methacrylate (Bis-GMA), bisphenol A ethoxylated dimethacrylate (Bis-EMA), and urethane dimethacrylate (UDMA) [11]. Exposure to TEGDMA, HEMA, or UDMA can produce reactive oxygen species (ROS) leading to cell damage [19, 20]. Further, the elevation of exposure levels can detrimentally induce DNA damage and cell death [21–23].

Considering the large variety of compositions in light-polymerised composites and their diverse usage in restorative dentistry, the potential risks of resin components leaching out into the oral cavity can be a significant concern to patients and practitioners. Therefore, it is meaningful that the biocompatibility of polymerised resins is assessed at the level of individual eluates both in quantitative and qualitative manners. Gas chromatography/mass spectrometry (GC/MS) has been used to identify additives, smaller monomers, comonomers and other volatile compounds, and decomposition and fragmentation products [24]. A strong correlation between the amount of eluates and cell viability was largely manifested using various cell lines and different test methods [22, 25–29]. Further, components released from polymerised composites can affect cellular signalling networks by generating ROS, in the same pattern as detected with cytotoxic effects [26]. Moreover, the crosslinking structures of matrix monomers and dispersed inorganic fillers can have mutual impacts on the reachability of unbound monomers, thus the filler contents are an interesting topic for investigating the toxicity of composite materials. However, limited information is available to dental practitioners when selecting the material considering safety issues in relation to leaching components of nanohybrid composites [13].

This study aimed to compare the chemical and biological characteristics of three nanohybrid dental composites by assessing filler particle analysis, monomer degree of conversion (DC), the composition of elutes, and cytotoxicity and ROS production in fibroblasts. The null hypothesis was that the components eluted from the three tested nanohybrid dental composites would show no differences in cytotoxicity or ROS production.

## Materials and methods

### Overview

**Fig. 1:**
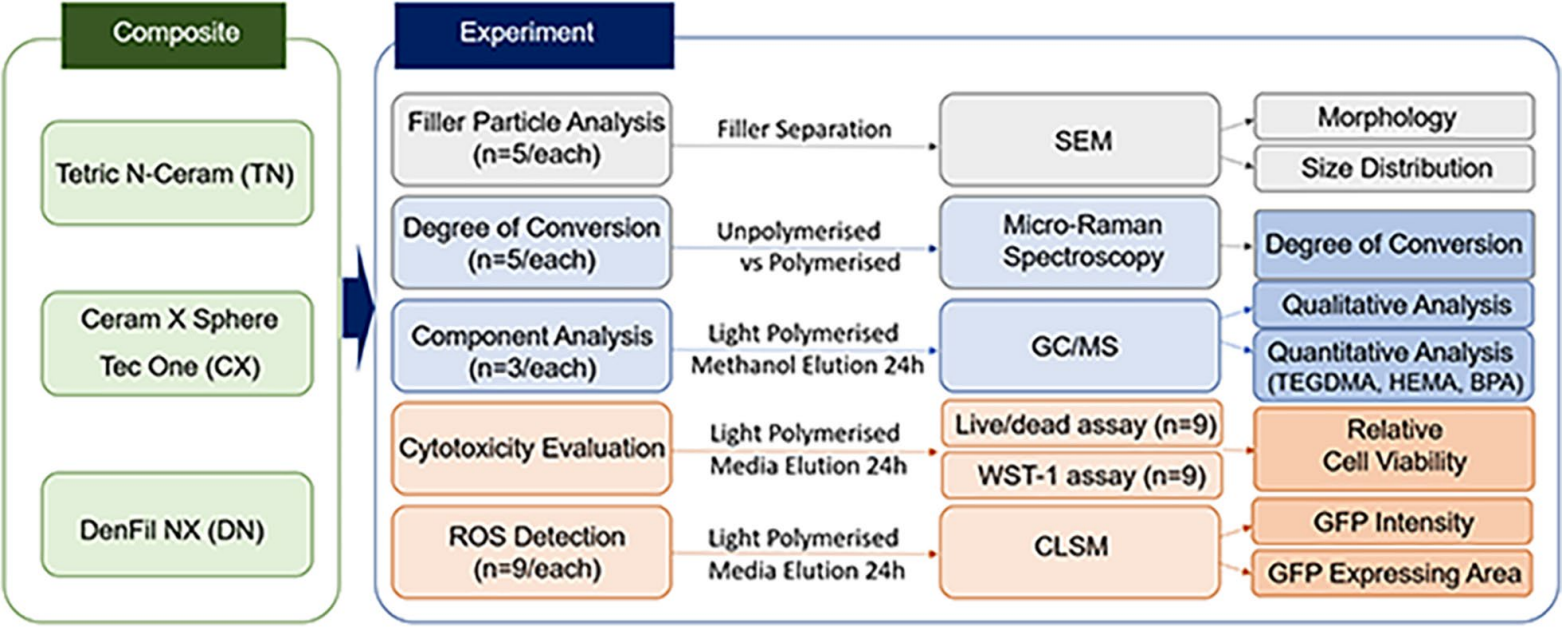
Experimental flow diagram

Three nanohybrid composite resins (Tetric N-Ceram, TN; Ceram X Sphere Tec One, CX; and DenFil NX, DN) were used in the study (Table 1). The variables of filler contents, DC, and eluate compositions were determined using scanning electron microscopy (SEM) analysis, micro Raman spectroscopy, and GC/MS. Live/dead and Water-Soluble Tetrazolium-1 assays were performed for cytotoxicity evaluation and real-time ROS production was assessed in fibroblasts (Fig. 1).

**Table 1:** Materials used in the study

Group	Type	Matrix composition	Filler composition	Filler degree (vol%, wt%)	Manufacturer (Lot No.)
TN	Nanohybrid	Bis-GMA, UDMA, Bis-EMA, TEGDMA	Barium aluminium glass (0.4 μm, 0.7 μm), Ytterbium trifluoride (0.2 μm), Mixed oxide (0.16 μm), Prepolymer	55–57, 76	Ivoclar Vivadent, Lichtenstein (Y50557)
CX	Nanohybrid ceramic	Bis-EMA, TEGDMA	The Sphere TEC fillers (15 μm), Nonagglomerated barium glass fillers (0.6 μm), Ytterbium fluoride (0.6 μm), Methacrylic polysiloxane, nanoparticles	59–61, 77–79	Dentsply Sirona, USA (2,009,000,471)
DN	Nanohybrid	Bis-GMA, UDMA, TEGDMA	Barium aluminosilicate (< 1 μm), Fumed silica (0.04 μm)	76–78, 81	Vericom, Korea (NX1601A2)

### Filler particle analysis

#### Filler particle preparation

For the filler particle characterization, the resin matrix of the composites (TN, CX, and DN) was dissolved and discarded to separate the fillers. A total of 300 mg of unpolymerised composites were placed in 10 mL amber glass vials, then immersed in 6 mL of acetone (99.5%, Sigma-Aldrich St. Louis, MO, USA) chloroform (99.8%, Sigma-Aldrich) and absolute ethanol (Sigma-Aldrich) successively and held for 24 h per each. While holding, the samples were centrifuged three times at 1,200 rpm for 6 min, and the supernatants were discarded. The remaining precipitation was dried overnight at 37 °C and sputter-coated for SEM observation.

#### SEM observation

The morphology and the size distribution of filler particles were observed under SEM (S-4700, FE-SEM, Hitachi, Tokyo, Japan) (*n* = 5). SEM images were taken at 10 kV, 0.1 nA, and a working distance of 10 mm at magnifications of 1,000, 5,000, and 30,000. The size and number of submicron-sized (smaller than 1 µm) were determined from 1,000 random particles at a magnification of 30,000 (*n* = 5). The spherical-shaped fillers were assessed from 100 random particles (*n* = 5) at 5,000 × and 30,000 x. ImageJ software (ver. 1.53, National Institutes of Health, Bethesda, MD, USA) was used for the image analysis [31, 32].

### Degree of conversion measurement

#### Specimen preparation

The composites (TN, CX, and DN) were prepared into disc-shaped specimens (13 mm diameter and 1 mm thickness) in a Teflon mould. Both top and bottom surfaces were cured for 40 s using a light-emitting diode (LED) curing unit with a wavelength of 430 – 480 nm and an intensity of 850 – 950 mW/cm^2^ (Elipar DeepCure-S LED Curing Light, 3 M ESPE, Seefeld, Germany). The top surface remained open to simulate the clinical situation of an oxygen-inhibited layer, while the bottom was covered with a glass slide.

#### micro-Raman spectroscopy

After light-polymerised, five points of each specimen (*n* = 5) were assessed with a 532 nm laser-equipped, micro-Raman microscope (DXR2xi, Thermo Fisher Scientific, Madison, WI, USA), showing a spectral resolution of approximately 5 cm^−1^ and a spectral range of 2000 – 1000 cm^−1^ at a magnification of 50. During the polymerisation, the intensity of the peak decreased with the conversion of the aliphatic double-carbon structure to form polymer chains. The following equation was used to calculate DC:

$${\text{DC }}\left( {\text{\% }} \right) = 1 - \frac{{{ }R_{Polymerised} }}{{R_{Unpolymerised} }} \times 100_{ }$$

where *R* is the ratio of peak intensity at 1639 cm^−1^ and 1609 cm^−1^ associated with the aliphatic and aromatic stretching in nanohybrid composites, respectively.

### Eluted component analysis

#### Sample preparation

To effectively achieve the maximum concentration of eluates, methanol (99.9%, Sigma-Aldrich) was used as an eluent. Three disc-shaped specimens (TN, CX, and DN) with 13 mm in diameter and 1 mm in thickness were fabricated in a Teflon mould and light-polymerised (Elipar DeepCure-S, 3M ESPE). The top surface remained open to simulate the clinical situation of an oxygen-inhibited layer, while the bottom was covered with a glass slide. The samples were immersed in methanol and eluted for 24 h at 37 °C in brown glass vials (3 cm^2^/mL).

#### GC/MS analysis

The eluted monomers and additives were qualitatively analysed using GC/MS (*n* = 3). A Trace Ultra GC Ultra gas chromatograph linked to a triple quadrupole mass spectrometer (TSQ 8000, Thermo) was used and transfused in the splitless mode. The compounds were separated using a GC column with geometry parameters of 60 m in length, 0.25 mm in diameter, and 0.25 μm in film thickness at a stationary phase with a split ratio of 1:10 and helium flowing at a constant rate of 1 mL/min. The GC oven was heated isothermally at 50 ℃ for 2 min, heated to 280 ℃ (25 ℃/min), held for 5 min, and then cooled to 250 °C. With an electron ionisation source temperature of 240 ℃, the mass spectrometer (MS) was set to the full scan mode, and data were recorded (mass range m/z 50 – 600) at 70 eV. For qualitative analysis, the relevant compounds were identified by comparing the retention time and mass spectra with their corresponding reference standards and the National Institute of Standards and Technology (NIST) library database [30]. The limit of quantification (LOQ) was in the range of 0.1 – 1,000 μg/mL.

For the quantitative analysis, the standard component of UDMA (Sigma-Aldrich), HEMA (Sigma-Aldrich), TEGDMA (Sigma-Aldrich), and BPA (Sigma-Aldrich) were calibrated (*n* = 3).

### Cytotoxicity and ROS evaluation

#### Preparation of composite eluates

For cytotoxicity evaluation and ROS detection, three disc-shaped specimens (TN, CX, and DN) with 13 mm in diameter and 1 mm in thickness were fabricated in a Teflon mould. The top surface remained open to simulate the clinical situation of an oxygen-inhibited layer, while the bottom was covered with a glass slide. The light-polymerised (Elipar DeepCure-S, 3 M ESPE) were immersed in Dulbecco's modified Eagle medium (DMEM, Hyclone, Logan, UT, USA) for 24 h at 37 ℃ in dark (ISO 10993–5:2009). After filtering with a 0.22 μm membrane filter unit (Corning Glass Works, Corning, NY, USA), the composite eluates were used for the following analysis.

#### Cell preparation

The human gingival fibroblast cell line (HGF-1, ATCC CRL-2014) was cultured with DMEM containing 1% penicillin (Gibco, Life Technologies, Grand Island, NY, USA), 1% streptomycin (Gibco), and 10% fetal bovine serum (Gibco) at 37 ℃ in a humidified chamber with 5% CO_2_. The cells were seeded in a 100 mm culture dish (SPL Life Sciences, Yeoju-si, Gyeonggi-do, Korea) and evaluated after the cells reached 80% confluence.

#### Live/dead and WST-1 assays

HGF-1 cells were seeded in a 35 mm confocal dish (SPL) at a concentration of 2 × 10^4^ cells/mL and incubated for 24 h. After incubation, cells were washed with phosphate-buffered solution (PBS, Gibco), treated with 1 mL of prepared composite eluates, and incubated for another 24 h. The negative control (NC) was treated with DMEM and the positive control (PC) was treated with 1 mM H_2_O_2_ (Sigma-Aldrich).

For live/dead assay, a Live/dead Viability kit (Invitrogen, Waltham, MA, USA) was used and observed under an inverted fluorescence microscope (DS-Ri2, Nikon Corporation, Tokyo, Japan) and a confocal laser microscope (LSM 700, Carl Zeiss, Thornwood, NY, USA). Live cells were observed with green fluorescence, and dead cells were observed with bright red fluorescence.

For WST-1 assay, the EZ-Cytox cell viability assay kit (DoGen Bio, Seoul, Korea) was used to determine cytotoxicity after 24 h of treatment. The optical density (OD) was determined at 450 nm using a microplate reader (AMR-100, Allsheng, Hangzhou, Zhejiang, China). Relative cell viability was calculated as the ratio of OD of experimental groups (TN, CX, and DN) to that of NC. The experiments were triplicated (*n* = 9).

#### Detection of ROS

HGF-1 cells were seeded in black, flat-bottom 96 well plates (Greiner Bio-One, Frickenhause, Germany) at a density of 1 × 10^4^ cells/mL and incubated for 24 h. After incubation, the medium was changed to 100 μL of the composites eluate-containing 5 μM CellROX green reagent (Life Technologies, Carlsbad, CA, USA) and observed at 37 °C for 16 h. Upon oxidation, the green reagent binds to DNA, and its signal is primarily localised in both the nucleus and mitochondria. To confirm the intracellular reaction, images and fluorescence intensity were recorded at 0, 4, 8, and 16 h using a multimode plate reader (Cytation 7, BioTek, Winooski, VT, USA). Green fluorescence was detected when the reagent was oxidised by ROS and then bound to DNA. The relative intensities and ROS-production areas of green fluorescent protein (GFP) were analysed by Gen5 software (ver. 1.9, Biotek). The experiments were triplicated (*n* = 9).

### Statistical analysis

Statistical significance was assessed using a t-test, one-way and two-way analysis of variance (ANOVA), followed by a Bonferroni multiple comparison test. Data were analysed using GraphPad Prism (ver. 9.0.0, GraphPad Software Inc., San Diego, CA, USA).

## Results

### Filler particle analysis

**Fig. 2:**
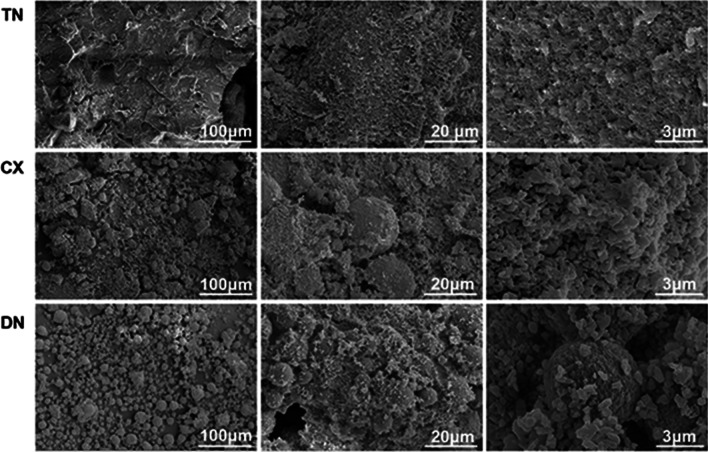
SEM images of agglomerated submicron-sized and spherical filler particles (magnification: left, 1,000 × ; centre, 5,000 × ; right, 30,000 ×). Submicron and nanofillers were aggregated to large spherical-shaped fillers (asterisk in CX and DN) that are dominant in CX and DN

**Fig. 3:**
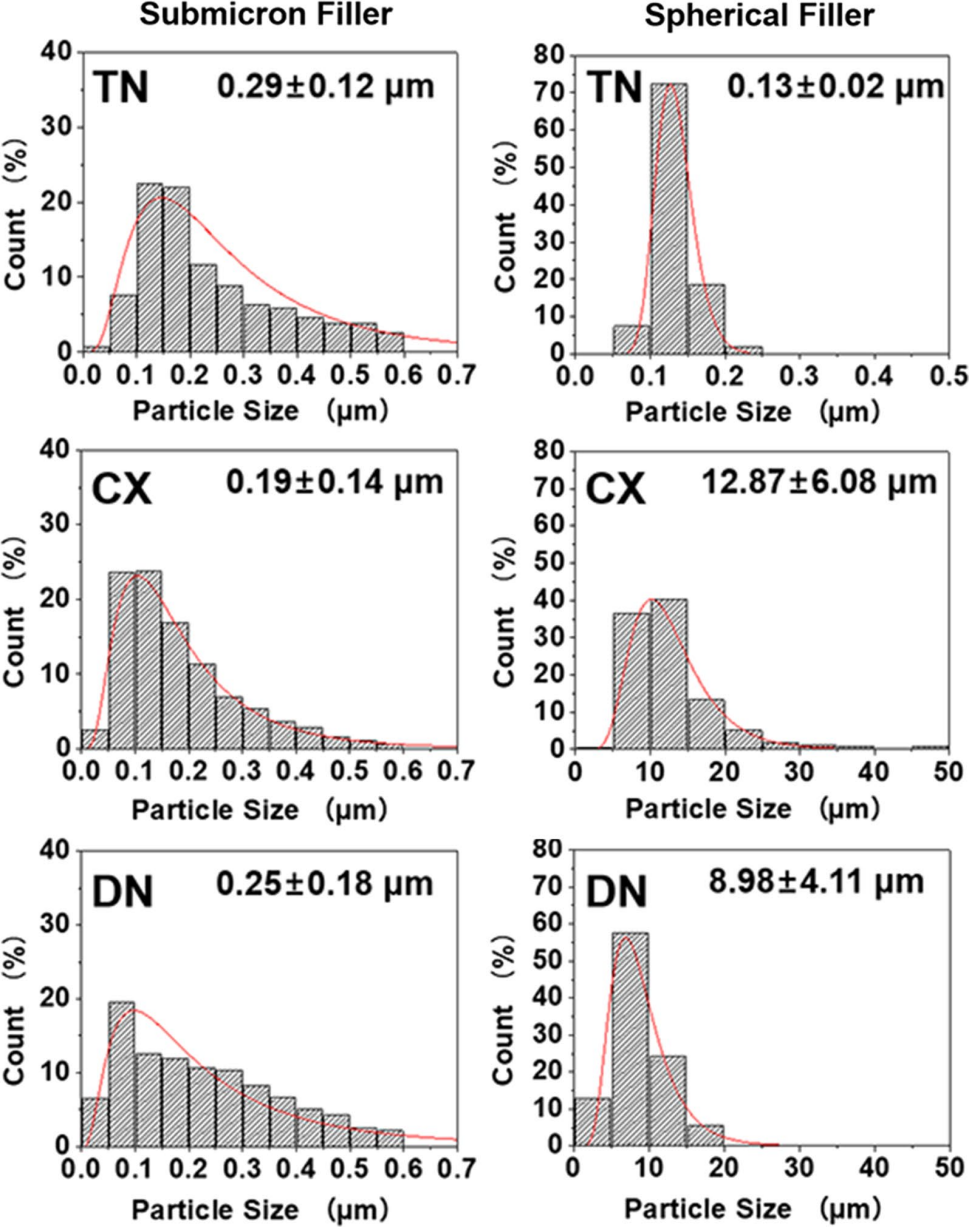
Size distributions of the filler particles. The average ± standard deviation of each filler is shown in the graph. CX and DN had larger spherical-shaped fillers than TN

The materials showed morphological variations of differently-sized fillers in SEM (Fig. 2). From the result of submicron filler distribution, particles smaller than 0.1 µm comprised 26.09% and 27.00% of the total distribution in CX and DN, while 7.63% in TN (Fig. 3). TN exhibited irregular submicron-sized fillers mixed with small spherical particles (0.13 ± 0.02 µm). In CX and DN, larger spherical particles (12.87 ± 6.08 µm and 8.98 ± 4.11 µm, respectively), were mixed with irregularly shaped fillers of submicron sizes (Table 2).

**Table 2:** Mean and standard deviation of the filler particle size (μm)

	Submicron-sized	Spherical-shaped
TN	0.29 ± 0.12^a^	0.13 ± 0.02^a^
CX	0.19 ± 0.14^a^	12.87 ± 6.08^b^
DN	0.25 ± 0.18^a^	8.98 ± 4.11^a^
*p*	0.5027	0.0014

Different superscripts indicate significant differences in a column (*p* < 0.05)

### DC measurement

**Fig. 4:**
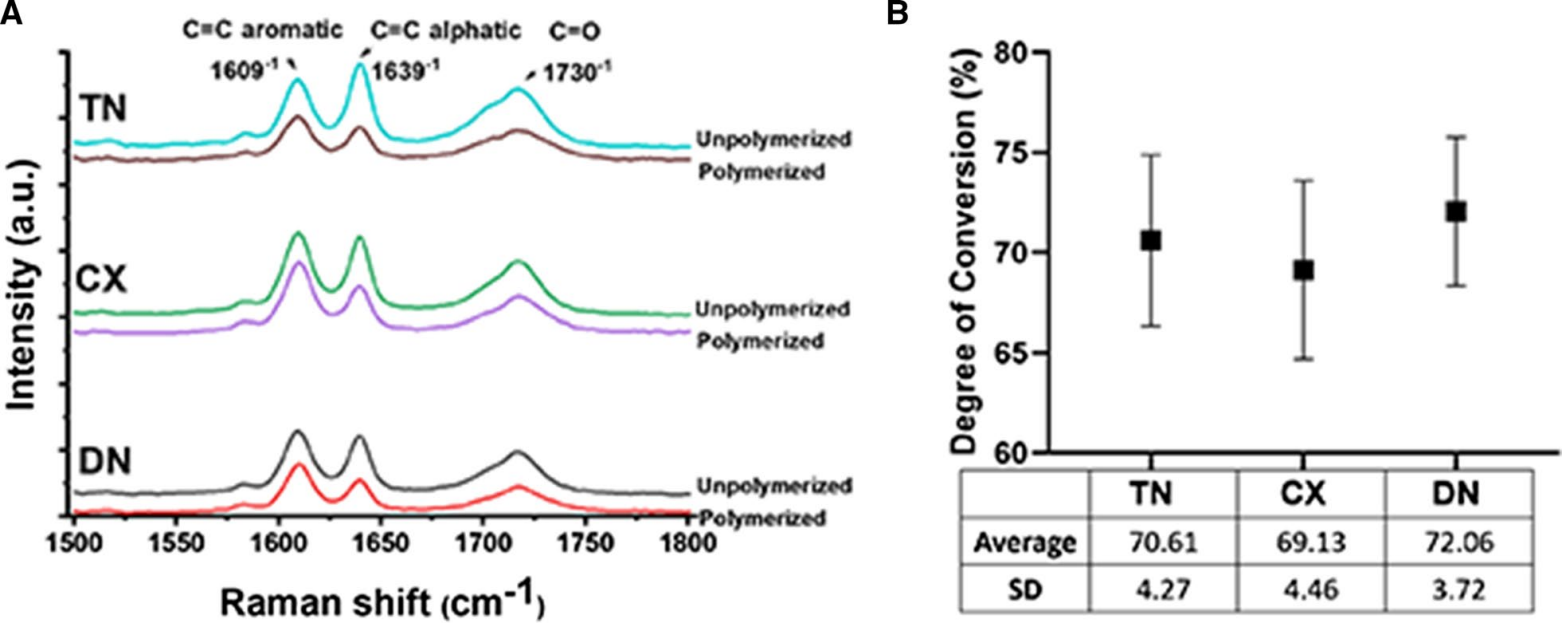
Degree of conversion. **A** Micro-Raman spectra. The intensities of 1609 cm^−1^, 1639 cm^−1^, and 1730 cm^−1^ were used to calculate the degree of conversion. **B** The degrees of monomer conversion was not significantly different among the groups (*p* > 0.05)

Micro-Raman spectra were obtained immediately before and after the light polymerisation (Fig. 4A). The peak intensities of 1609 cm^−1^ at 1639 cm^−1^ were associated with C=C aliphatic and aromatic stretching bonds in a polymeric matrix, respectively. The DC values in TN (70.61 ± 4.27), CX (69.13 ± 4.46), and DN (72.06 ± 3.72) were not significantly different (*p* = 0.05545, Fig. 4B).

### Qualitative and quantitative analysis of composite elutes

**Fig. 5:**
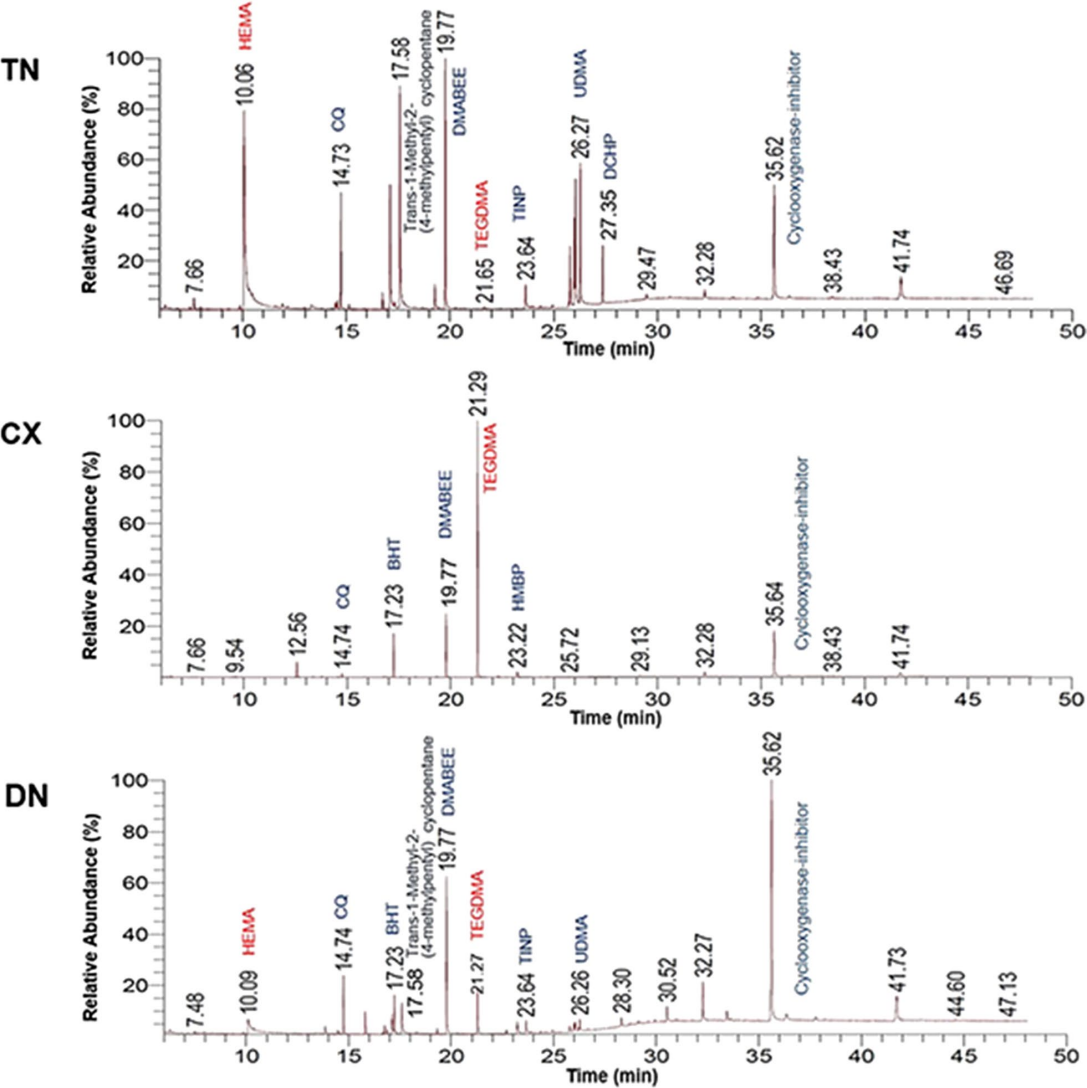
GC/MS chromatogram of the qualitative analysis. Differences in components and relative abundance of monomers and additives in TN, CX, and DN were confirmed

**Fig. 6:**
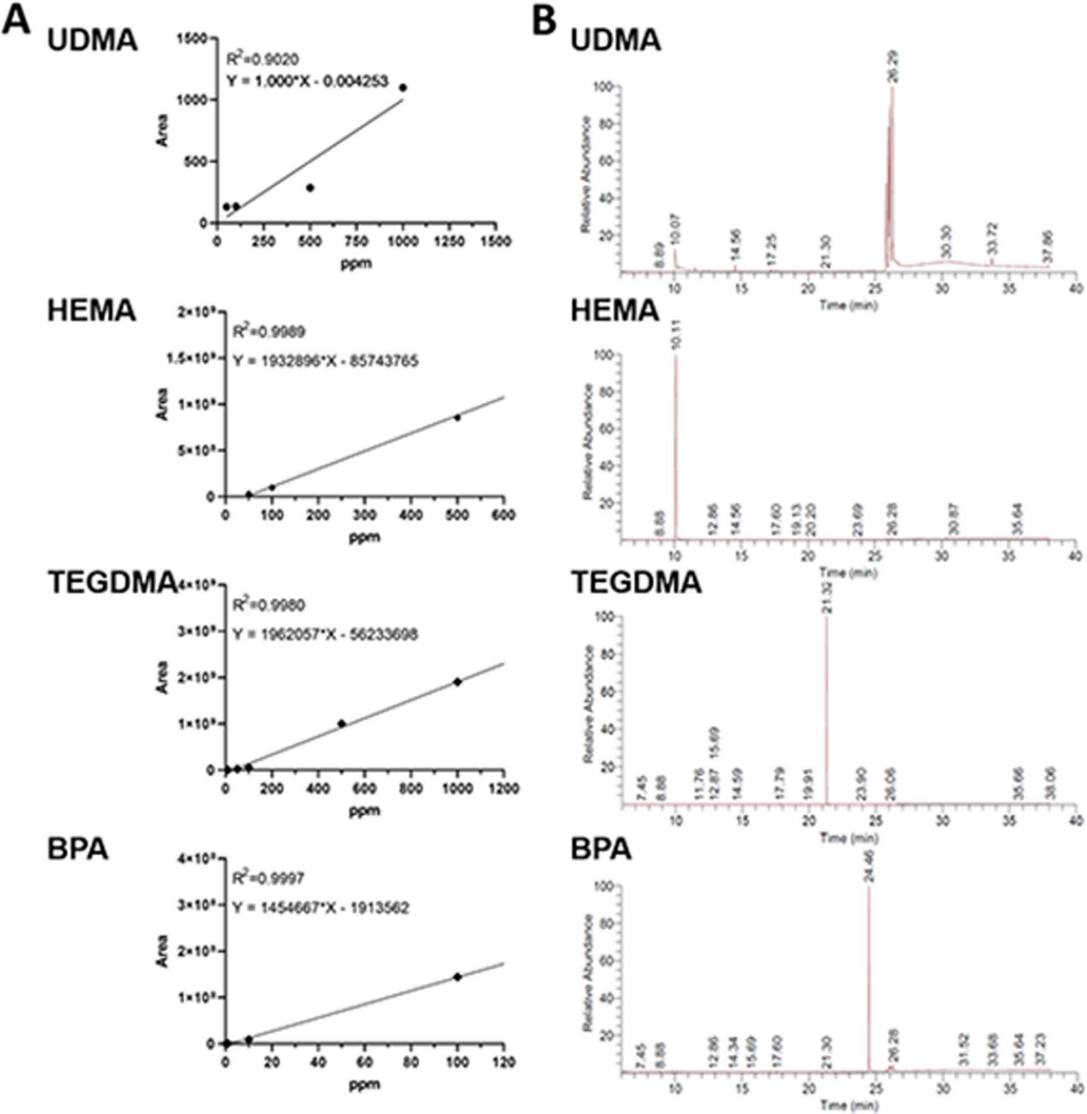
Calibration of selected standard components (UDMA, HEMA, TEGDMA, and BPA). **A** Calibration curves. The equations were used for the quantitative analysis. **B** Relative abundance of the standard components

Representative GC/MS chromatograms of individual substances in TN, CX, and DN were depicted (Fig. 5). HEMA was detected in both TN and DN. TEGDMA, camphorquinone (CQ), and the co-initiator, 4-dimethylaminobenzoic acid ethyl ester (DMABEE) were detected in all composites. Detected components of co-monomers and other additives are listed in Table 3. Calibration curves are obtained (Fig. 6A) and the qualification analysis of standard components is presented (Fig. 6B). In UDMA, due to multiple peaks depicted in GC/MS and non linear correlation coefficient (*R*^2^ = 0.9020), the quantitative analysis was not performed with UDMA (Table 4).

**Table 3:** Qualitative analysis by GC/MS

Compound name	Function	Formula	Molecular weight	t_R_ (min)	Area%		
					TN	CX	DN
2-Hydroxyethyl methacrylate (HEMA)	Monomer	C_6_H_10_O_3_	130.14	10.06	21.70 ± 0.30	–	4.46 ± 0.15
Triethylene glycol dimethacrylate (TEGDMA)	Monomer	C_14_H_22_O_6_	286.32	21.65	0.26 ± 0.10	48.87 ± 3.90	3.77 ± 0.39
Camphorquinone (CQ)	Photoinitiator	C_10_H_14_O_2_	166.22	14.73	4.36 ± 0.58	0.78 ± 0.12	4.47 ± 0.18
4-Dimethyl amino benzoic acid ethyl ester (DMABEE)	Co-initiator	C_11_H_15_NO_2_	193.24	19.77	9.02 ± 1.65	12.93 ± 1.85	13.49 ± 0.21
Benzyl iodide	Others	C_7_H_7_I	218.03	12.55	–	3.81 ± 0.77	–
Butylated hydroxytoluene (BHT)	Inhibitor	C_15_H_24_O	220.35	17.23	–	8.82 ± 2.64	3.69 ± 1.61
Trans-1-methyl-2-(4-methylpentyl) cyclopentane	Others	C_12_H_24_	168.32	17.58	11.68 ± 0.27	–	4.14 ± 0.31
2-Hydroxy-4-methoxybenzophenone (HMBP)	UV-absorber	C_14_H_12_O_3_	228.24	23.22	–	2.02 ± 0.39	–
2-(2-Hydroxy-5-methylphenyl) benzotriazole (TINP)	UV-stabilizer	C_13_H_11_N_3_O	225.25	23.64	1.71 ± 0.21	–	1.49 ± 0.11
2-(3'-Hydroxy-4'-methoxyphenyl)-5-methoxy-3-(3",4",5-trimethoxyphenyl) benzofuran-6-o	Cyclooxygenase-inhibitor	C_25_H_24_O_8_	452.45	35.62	10.35 ± 1.55	15.85 ± 1.39	33.59 ± 1.08
4,4'-[5-(1,1-Dimethylethyl)-2-methoxy-1,3-phenylene] bisdibenzofuran	Others	C_35_H_28_O_3_	496.59	41.74	3.61 ± 0.90	1.67 ± 0.15	5.89 ± 0.07
Dicyclohexyl phthalate (DCHP)	Plasticizer	C_20_H_26_O_4_	330.42	27.35	2.46 ± 0.18	–	–

**Table 4:** Quantitative analysis of HEMA, TEGDMA, and BPA. Data are presented as the mean and standard deviation in μg/mL (mM)

	HEMA	TEGDMA	BPA
TN	259.46 ± 53.14^a^ (1.99 ± 0.41)	23.3 ± 0.06^a^ (0.08 ± 0.00)	Not detected
CX	Not detected	1081.10 ± 128.60^b^ (3.77 ± 0.45)	Not detected
DN	43.91 ± 3.32^b^ (0.34 ± 0.02)	38.80 ± 3.50^a^ (0.13 ± 0.12)	Not detected
*p*	0.0022^*^	< 0.0001	–

^*^Result of the unpaired *t*-test between TN and DN

Different superscripts indicate significant differences in a column (*p* < 0.05)

The amount of HEMA in TN (259.46 ± 53.14 μg/mL) was significantly higher than that of DN (43.91 ± 3.32 μg/mL, *p* = 0.0022), and it was not detected in CX (Table 4). The amount of TEGDMA (1,081.10 ± 128.61 μg/mL) in CX was higher than those of TN and DN (23.3 ± 0.06 μg/mL, *p* < 0.0001 and 38.80 ± 3.50 μg/mL, *p* < 0.0001, respectively). BPA was not detected in any composite eluates.

### Cytotoxicity evaluation

**Fig. 7:**
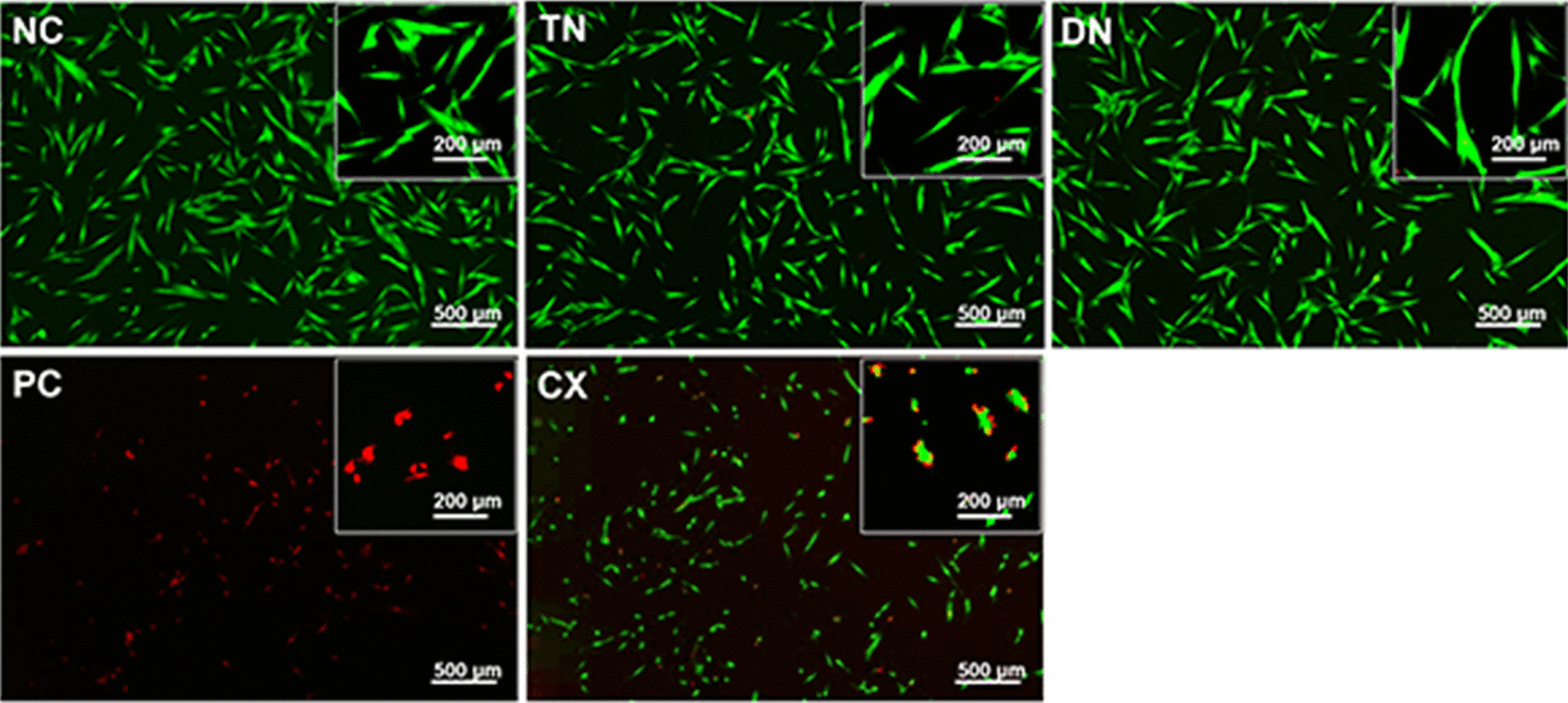
Representative images of live/dead assay. The merged fluorescent images with viable cells appear in green and dead cells in red. Dead cells and shrinkage of the cells were apparent in CX compared with NC, TN, and DN. TN and DN showed a slight decrease in cell density compared to NC

From the fluorescence images, TN and DN showed a slight decrease in cell density without apparent changes in cell morphology compared to NC (Fig. 7). In CX, dissimilar cell morphology with less homogeneity with shrunken cellular processes was observed.

**Fig. 8:**
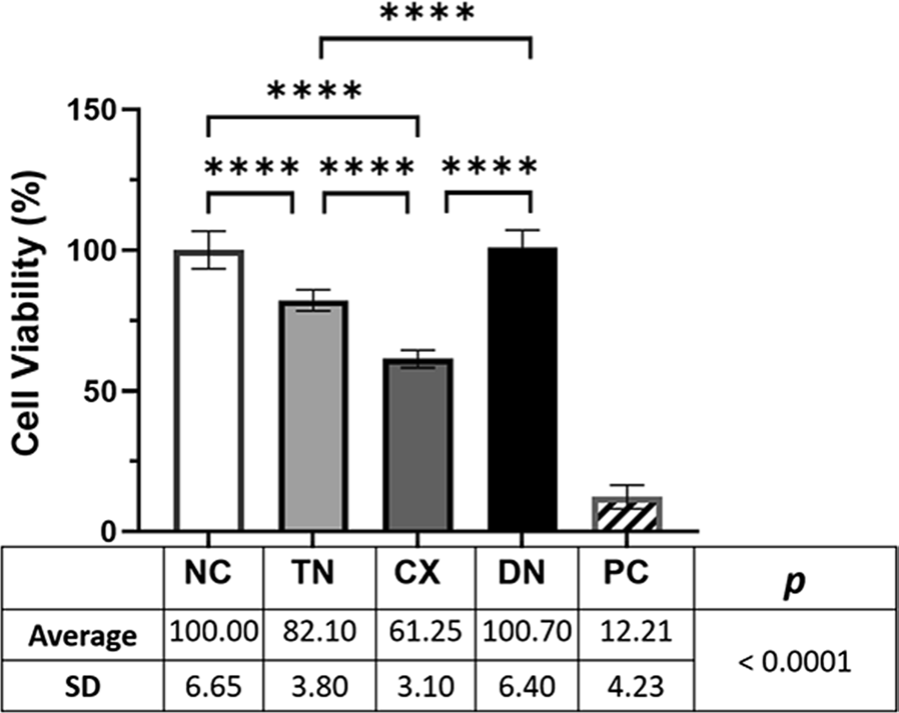
Relative cell viability by WST-1 assay. TN and CX showed significantly decreased cell viability compared with NC and DN (****p < 0.0001). NC and DN showed no significant difference after 24 h

The relative cell viability was the highest in DN (100.70 ± 6.40) with no significant difference compared to NC (Fig. 8). TN (82.10 ± 3.80) and CX (61.25 ± 3.10) showed a significant decrease in cell viability (*p* < 0.0001).

### Detection of ROS

**Fig. 9:**
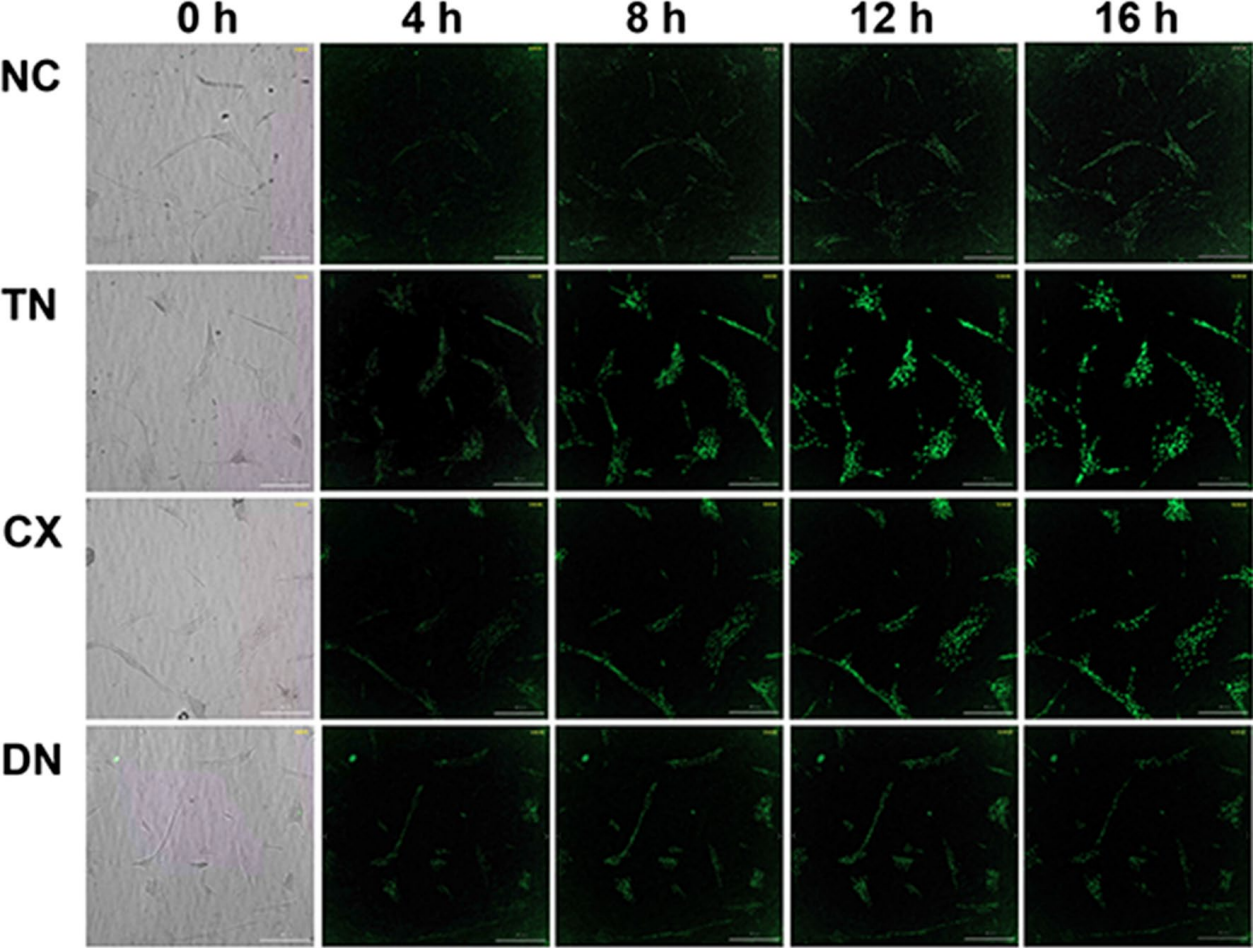
Representative images of real-time ROS (4 ×). The fluorescence intensity was observed for 16 h after the treatment with composite eluates. The green fluorescence was more evident in TN and CX compared to NC

**Fig. 10:**
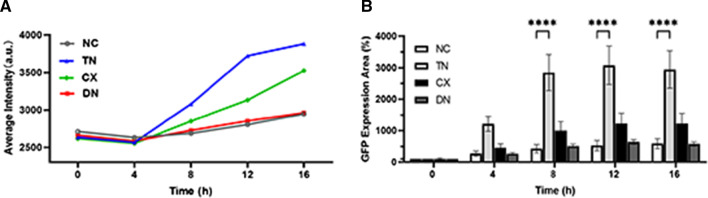
Fluorescence intensity and expression area of GFP. **A** Relative intensities of green fluorescence at each time interval. After 8 h, significantly higher intensities were detected in TN and CX compared to NC. **B** Relative GFP expression areas were calculated using relative values at zero hours. TN showed significant increases at 8 h, 12 h, and 16 h compared to NC. (****p < 0.0001)

Fluorescent digital image correlation micrograph profiles exhibited a time-dependent increase in ROS generation (Fig. 9 and Additional file 1: Video S1). The relative intensity significantly increased in TN and CX from 8 h compared with NC (*p* < 0.0001) (Table 5 and Fig. 10A). The relative GFP expression area was calculated by relative values at zero hours (Table 6). The area in TN showed the highest ROS production followed by CX, those values were significantly higher than those in NC and DN (*p* < 0.0001) (Fig. 10B). DN showed no significant increase in ROS compared to NC until 16 h.

**Table 5:** GFP expression intensities in mean ± standard deviation

	0 h	4 h	8 h	12 h	16 h
NC	2712 ± 74^a^	2632 ± 70^a^	2687 ± 26^a^	2805 ± 82^a^	2946 ± 180^a^
TN	2633 ± 219^a^	2567 ± 145^a^	3080 ± 61^b^	3723 ± 119^b^	3825 ± 120^b^
CX	2618 ± 59^a^	2554 ± 41^a^	2851 ± 34^c^	3132 ± 104^c^	3526 ± 158^c^
DN	2663 ± 46^a^	2585 ± 28^a^	2727 ± 93^a^	2853 ± 93^a^	2962 ± 50^a^
*p*	0.3846	0.2395	< 0.0001	< 0.0001	< 0.0001

Different superscripts indicate significant differences in a column (*p* < 0.05)

**Table 6:** Relative GFP expression area in mean ± standard deviation (%)

	0 h	4 h	8 h	12 h	16 h
NC	100.00 ± 0.00^a^	273.36 ± 253.63^a^	428.27 ± 401.26^a^	527.69 ± 484.40^a^	587.12 ± 480.53^a^
TN	100.00 ± 0.00^a^	1214.73 ± 723.05^a^	2848.86 ± 1721.45^b^	3075.96 ± 1836.66^b^	2945.94 ± 1779.69^b^
CX	100.00 ± 0.00^a^	465.22 ± 355.18^a^	1005.96 ± 844.07^a^	1229.27 ± 978.90^a^	1227.90 ± 958.57^a^
DN	100.00 ± 0.00^a^	271.90 ± 113.32^a^	516.21 ± 174.13^a^	642.89 ± 229.58^a^	577.71 ± 192.06^a^
*p*	> 0.0999	> 0.0999	< 0.0001	< 0.0001	< 0.0001

Different superscripts indicate significant differences in a column (*p* < 0.05)

## Discussion

Based on the results, three nanohybrid composites revealed different compositions of monomers and additives. HEMA and TEGDMA, representative co-monomers with low molecular weights and high mobility were eluted with different amounts in three composites, affecting the cell viability and ROS production to dissimilar levels. Therefore, our null hypothesis was rejected.

Currently, commercially available nanohybrid composites enhanced filler loading and also replaced conventional monomer-matrix formulations to maintain adequate consistency and aesthetic properties. It is common that manufacturers only indicate the total volume and weight of filler contents. Additionally, the remaining volume is occupied with resin matrix monomers and other trace additives that users are unaware of. This study focused on co-monomers of low molecular weight, which increase the polymerising effectiveness and manipulating efficiency, rather than backbone monomers of high molecular weight with elevated mechanical and chemical stabilities. Regarding the cytotoxicity of monomers released from restorative composites, the intensity of cytotoxicity of monomers was ranked as Bis-GMA > UDMA > TEGDMA > HEMA [33, 34]. However, the order of releasing tendency is known to be HEMA > TEGDMA > UDMA > BisGMA, signifying an elution capacity of small-sized monomers [11]. For methacrylate cross-linking monomers, differences exist in the magnitude of released quantities between the organic solvent and water-based solution [29]. The GC/MS experimental method is based on the vaporization and ionization of ingredients of low molecular weight compounds. And methanol was chosen to meet the limit of quantified detection due to its major dissolution efficiency. In the results, CX showed the highest TEGDMA level and had the lowest cell viability, as well as a time-dependent increase in ROS production. As for HEMA, the value detected in TN (1.9 mM) was higher than in DN (0.34 mM) and CX (non-detect). It was widely reported that HEMA could be a degradation product from UDMA as being a basic monomer with high molecular weight [35]. UDMA is also a basic component in the TN, as claimed by the manufacturer, and the higher concentration of HEMA detected in TN was regarded to be derived from UDMA. In our GC/MS, a standard UDMA (≥ 97%, Sigma-Aldrich, Cat no. 72869–86-4) was analysed, and the peaks were confirmed as four single peaks and one single peak of HEMA (Fig. 6B). In a previous study involving monomer releases from dentin bonding systems, when using methanol to extract the resin components, the mean content of HEMA in methanol was 10 times higher than that in distilled water [36]. Upon oxidation, the cell ROX green reagent binds to DNA and thus, its signal is primarily localised in the nucleus and mitochondria. The EC_50_ values of HEMA for the viability of HGFs were 11.2 mM and the concentration inducing DNA strand breakdown was 1.12 mM [14]. Despite being overly extracted in methanol, HEMA was prominently detected in the TN group and might have an impact on the generation of ROS. Conventionally, BPA is a component that has been at the centre of debates due to its xeno-estrogenic potential, resulting in systemic consequences. However, many previous studies that applied highly sensitive analytical methods did not reveal the presence of BPA, despite the possible presence as an impurity during manufacturing. Biodegradation of BPA-based basic monomers into BPA was also feasible under the extremes of the oral environment such as pH fluctuation, enzymatic degradation, and thermal and mechanical challenges. Still, only trace amounts could be detected under hazardous limits, particularly with the experimental settings for short-term elution [18]. Even with methanol as an immersion medium to dissolve extractable compounds, we did not detect BPA in the eluates from the three composites.

In our study, the level of DC reached around 70% without significant differences among the composite. Therefore, the differences in the releases of unpolymerised monomers seemed not to result from different degrees of polymerisation of composites. It was interesting that no significant cytotoxicity or ROS generation was revealed in the DN group. Apart from the relatively low elution of TEGDMA and HEMA in DN, the volumetric content of fillers is higher in DN than in others (Table 1). We assumed that the lower content of matrix monomer in DN might contribute to the smaller amount of monomer release, resulting in better biocompatibility. Another point to consider for the relatively lower level of cytotoxicity detected in DN is that DN does not contain any fluoride compounds that are claimed to be the contents of TN and CX. Previous studies showed that even novel composites containing synthetic fillers conjugated with fluoride ions revealed significant levels of anti-cariogenic potential, but not any detectable level of cytotoxicity [37, 38]. However, it will be meaningful to investigate the ionic release capacity of the composites aside from their monomer elution.

Regarding our limited experimental designs, it is hard to extrapolate the results to clinical circumstances. Since restorative composite resins are composed of various compounds, no single detection method can achieve the evaluation of every compound with various molecular weights and chemical formulas. We will need extended chemical analysis to evaluate monomers of higher molecular weights such as Bis-GMA and UDMA [39]. The selection of the immersion medium is a complicated issue. Even when human saliva is used, the thermal, chemical, and bacterial conditions need to be incorporated to assimilate in vivo circumstances [40]. To simulate salivary flushing in the mouth, constant exchange of immersion medium also needs to be considered. In addition, mechanical impacts on restorative surfaces during intraoral service of dental composites should be considered, since dislodgement of surface fillers from the matrix can be developed, accelerating monomer elution [41]. Future studies need to investigate more clinically relevant conditions and reflect those factors in experimental settings.

## Conclusion

Based on this in vitro study, three nanohybrid dental composites exhibited various compositions of filler sizes and resin components, resulting in different levels of cytotoxicity and ROS production. Chemical compositions of dental composites can be considered with their biological impact on safety issues in the intraoral use of dental restorative composites. The composite eluate (CX) with the highest TEGDMA showed the highest cytotoxicity induced by ROS accumulation. The composites eluate (DN) with the lower TEGDMA and HEMA presented the highest cell viability.

## Supplementary Information

**Additional file 1: video S1** Real-time ROS detection in HGF-1. Nanohybrid composites eluate results in a time-dependent increase in ROS production in HGF-1.

## Data Availability

The data and materials used and/or analysed during the current study are available from the corresponding author upon reasonable request.

## Abbreviations

ROS: Reactive oxygen species
MAPK: Mitogen-activated protein kinase
DC: Degree of conversion
Area%: Peak area in percentage
HEMA: 2-Hydroxyethyl methacrylate
TEGDMA: Triethylene glycol dimethacrylate
CQ: Camphorquinone
DMABEE: 4-Dimethyl amino benzoic acid ethyl ester
BHT: Butylated hydroxytoluene
HMBP: 2-Hydroxy-4-methoxybenzophenone
TINP: 2-(2-Hydroxy-5-methyl phenyl) benzotriazole
DCHP: Dicyclohexyl phthakate
